# Supplementary material for: Improving the Inhibitory Effect of Phages against Pseudomonas aeruginosa Isolated from a Burn Patient Using a Combination of Phages and Antibiotics
Source: Viruses. 2021 Feb 21;13(2):334. doi: 10.3390/v13020334 (PMC7926668; doi:10.3390/v13020334)
Supplement: Supplementary file 1 [file viruses-13-00334-s001.zip › viruses-1097653-suppl/Revised-Supplementary-Aghaee-KhanMirzaei et al.pdf]

Figure S1. Whole genome alignments of phages 6, 32 and 45. A) compares the similarity between all three phages in the study that showed less than 1% pairwise identity on nucleotide level. B) shows 93% homology over 79% coverage between 6 and vB_PaeM_C1-14_Ab28. C) shows 97% genetic similarities over 58% coverage between 45 and vB_PaeM_C2-10_Ab02. D) compares genetic homology of phages 32 and PA02 at a 94.4% pairwise identity over 54% of the genome.

Table S1. Efficiency of plating (EOP) for the four selected phages on 12 strains of *P. aeruginosa* isolates. The EOP was calculated by dividing the PFU on target bacteria by PFU on host bacteria. NP: no plaque was detected; -: the original hosts.

Table S2. Effect of Gentamicin and Ciprofloxacin on prophage induction. The impact of both antibiotics, at different MICs and incubation times, on prophage induction of *P. aeruginosa* strain #14. For each antibiotic, concentration, and incubation time condition, infectious induced phages were detected using spot assay on different *P. aeruginosa* strains. 0: no phages; 1: phage detected. Ten *P. aeruginosa* strains listed in the table were used as potential hosts to detect induced phages from *P. aeruginosa* isolate #14. Results are from three biological replicates.
