# Supplementary figures and images for: Improving the Inhibitory Effect of Phages against Pseudomonas aeruginosa Isolated from a Burn Patient Using a Combination of Phages and Antibiotics
Source: Viruses. 2021 Feb 21;13(2):334. doi: 10.3390/v13020334 (PMC7926668; doi:10.3390/v13020334)

**A**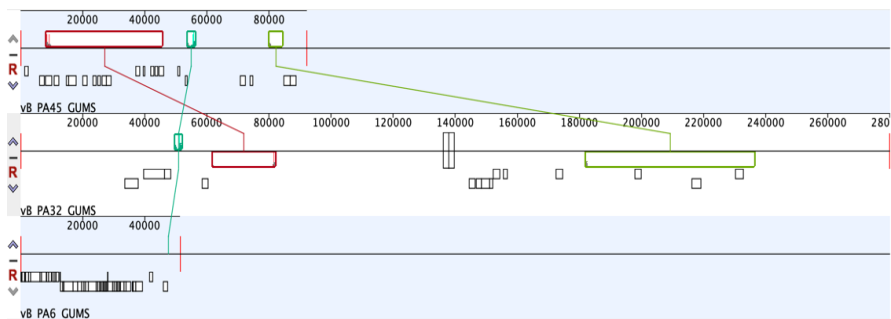**B**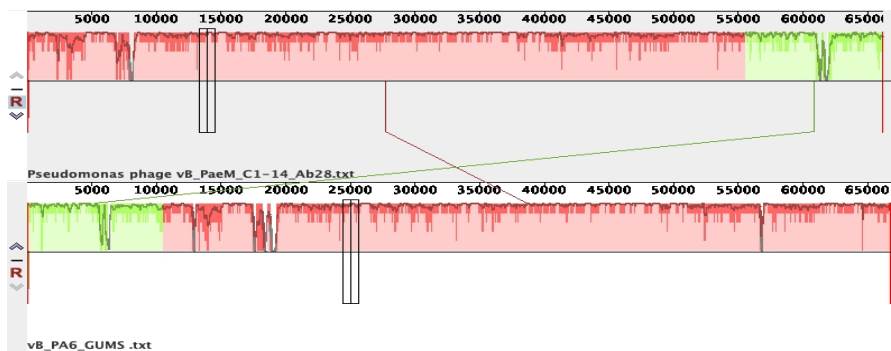**C**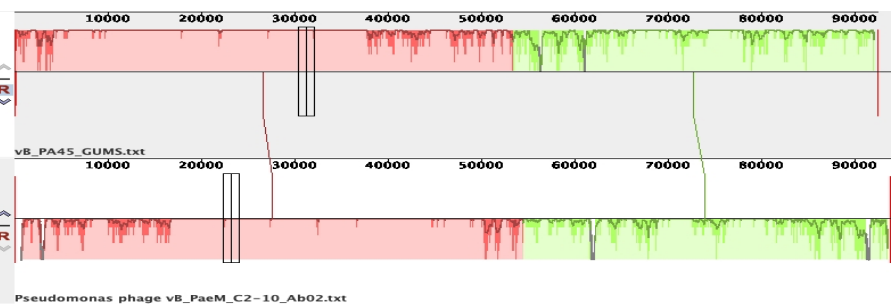**D**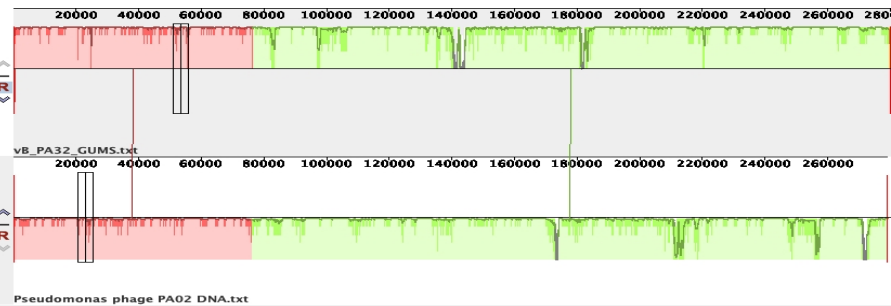

Supplement: Supplementary file 1 [file viruses-13-00334-s001.zip › viruses-1097653-suppl/Figure S1.pdf]
